# Structural perspectives on chemokine receptors

**DOI:** 10.1042/BST20230358

**Published:** 2024-06-10

**Authors:** Kanwal Kayastha, Yangli Zhou, Steffen Brünle

**Affiliations:** Leiden Institute of Chemistry, Faculty of Science, Leiden University, Einsteinweg 55, Leiden 2333 CC, The Netherlands

**Keywords:** allosteric regulation, biased signaling, chemokine receptor, G-protein-coupled receptors, receptor activation

## Abstract

Chemokine receptors are integral to the immune system and prime targets in drug discovery that have undergone extensive structural elucidation in recent years. We outline a timeline of these structural achievements, discuss the intracellular negative allosteric modulation of chemokine receptors, analyze the mechanisms of orthosteric receptor activation, and report on the emerging concept of biased signaling. Additionally, we highlight differences of G-protein binding among chemokine receptors. Intracellular allosteric modulators in chemokine receptors interact with a conserved motif within transmembrane helix 7 and helix 8 and exhibit a two-fold inactivation mechanism that can be harnessed for drug-discovery efforts. Chemokine recognition is a multi-step process traditionally explained by a two-site model within chemokine recognition site 1 (CRS1) and CRS2. Recent structural studies have extended our understanding of this complex mechanism with the identification of CRS1.5 and CRS3. CRS3 is implicated in determining ligand specificity and surrounds the chemokine by almost 180°. Within CRS3 we identified the extracellular loop 2 residue 45.51 as a key interaction mediator for chemokine binding. Y291^7.43^ on the other hand was shown in CCR1 to be a key determinant of signaling bias which, along with specific chemokine-dependent phosphorylation ensembles at the G-protein coupled receptors (GPCR's) C-terminus, seems to play a pivotal role in determining the direction of signal bias in GPCRs.

## Introduction

G-protein coupled receptors (GPCRs) are a large family of human membrane receptors that fulfill viable roles in many physiological processes, such as vision, taste, smell, and immune responses. GPCRs transduce extracellular signals towards the intracellular lumen and contribute in this way to cell communication within the human body. Due to their vital function and association with many human diseases, GPCRs are prime targets for drug discovery. Structural investigations of GPCRs have played a significant role in the last decade in elucidating the molecular mechanism of receptor activation, function, and regulation. This review focuses on the current structural knowledge of a specific family of class-A GPCRs called chemokine receptors. Chemokine receptors constitute together with Chemerin -, Glycoprotein hormone -, and prokineticin receptors a class-A subgroup whose natural ligands are proteins. Chemokine receptors can be classified into CCR, CXCR, CX3CR, and XCR receptors, according to the first two N-terminal cysteines within the respective chemokine ligand (CCL, CXCL, CXC3L, XCL). Additionally, atypical chemokine receptors (ACKR) belong to the chemokine receptor group that do not couple to G-proteins and are thought to function as scavenger receptors. Chemokines, the natural ligands of chemokine receptors are small proteins (8–15 kDa) that belong to a family of small cytokines that are mostly associated with the directional movement of leukocytes and other cell types within immune responses. Therefore, chemokine receptors play a major role in the context of human immunity and are central to the process of immune cell migration and localization of leukocytes throughout the human body.

A total of 48 chemokines are documented in humans that interact with 23 chemokine receptors; therefore, the majority of receptors respond to multiple chemokines and most chemokines bind to multiple receptors, constructing a seemingly redundant system at first glance. However, recent insights into chemokine signaling suggest a highly fine-tuned and tightly regulated system, with spatial and temporal control in chemokine expression [1,2]. Because of the complexity of the chemokine system and its vital function for the human body, chemokine receptors have been implicated in a plethora of immune and inflammatory-related disorders, including arthritis, diabetes, and inflammatory bowel disease [3]. The particularly strong involvement in all stages of cancer development and spreading, like the promotion of angiogenesis and metastasis, makes these receptors a spotlight for academic and industrial research and drug discovery efforts [4].

**Figure 1.**
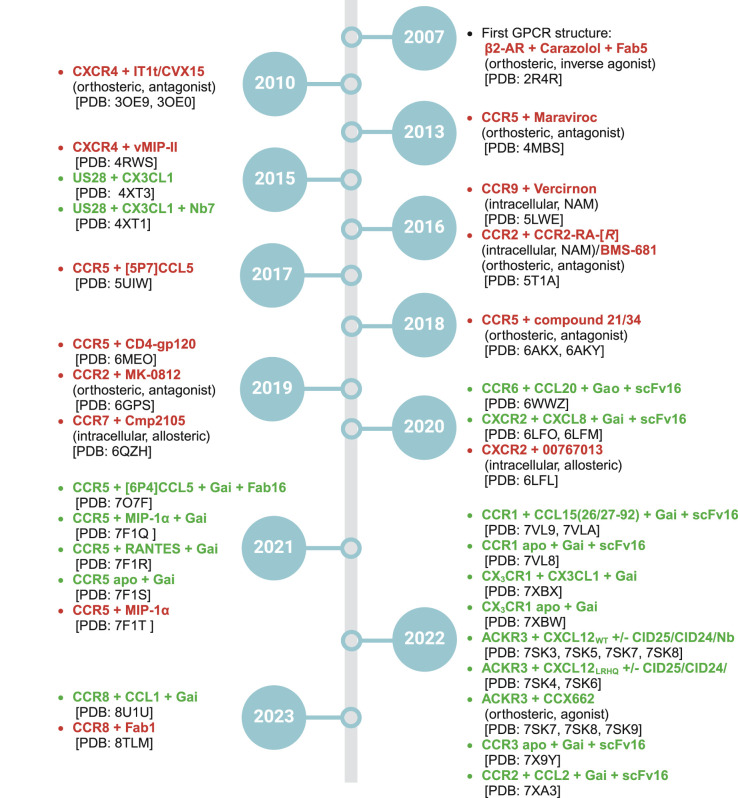
Timeline of the structural elucidation of chemokine receptors. In 2007 the first GPCR structure was solved that showed the β2-adrenergic receptor (β2-AR) bound to the inverse agonist Carazolol in an inactive conformation (red) via X-ray crystallograllography. This structure was followed by a range of structures in an inactive conformation including the first chemokine structure in 2010 that showed CXCR4 bound to the orthosteric antagonists IT1T and CVX15 (structures with bound small molecules are shown with additional information about location and mode of action). 2020 marked the first year in which structural information of a chemokine receptor in a true active state was solved (green) via single-particle cryo-electron microscopy. This led to a plethora of active structure that investigated the chemokine recognition mechanisms as well as the nuances of biased signalling. The entries of the viral chemokine receptor US28 were added for completeness.

The current understanding within the field of chemokine receptor research is shaped by significant milestones of structural elucidations. Figure 1 offers a chronological overview of these developments which is accompanied by a description in the supplementary information that highlights key findings of each structure.

In this review, we highlight the current state of knowledge revealed by the structural analysis of chemokine receptors. We discuss negative allosteric modulation, chemokine receptor activation mechanisms, biased signaling in chemokine receptors, and intracellular G-protein binding mechanisms.

## Insights from inactive chemokine receptor structures

### Allosteric modulation of chemokine receptors via the intracellular allosteric binding site

Historically, classical GPCR drug discovery focused on the orthosteric extracellular binding pocket with the aim to block the binding for natural ligands and thus prevent the signaling cascade from emerging. However, in environments with high concentrations of endogenous ligands, such as during inflammation, orthosteric binders compete with natural ligands for the binding site and therefore often lack the necessary efficacy for the intended receptor [5]. Importantly, orthosteric binding sites share high sequence homologies among their receptor subtypes, leading to off-target effects. The main reasons for drugs to fail in Phase II and Phase III clinical trials are attributed to safety or efficacy concerns [6,7]. In contrast, allosteric ligands usually bind to spatially and, more importantly, topologically distinct (allosteric) sites on the receptor [8,9] that are generally less conserved among receptor subtypes and therefore offer unparalleled subtype selectivity. Allosteric modulators can alter receptor activity either positively (PAM), negatively (NAM), act as silent modulators (SAM), or can have agonistic activity coupled to their allosteric modulation (Ago-PAM/Ago-NAM) [10]. Allosteric sites on GPCRs have been shown to be vastly different in their topology, ranging from binding sites on the extracellular vestibule [11] to bindings sites outside of the transmembrane bundle [12,13], as well as on the intracellular side [14,15].

**Figure 2.**
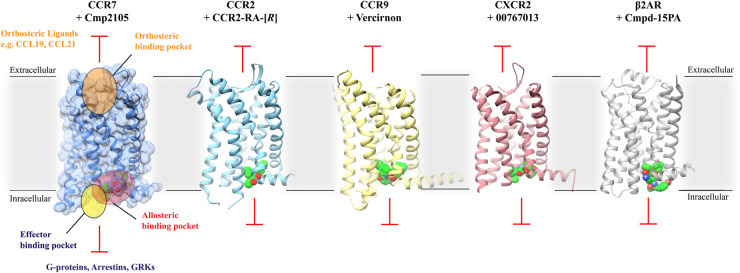
Overview of the X-ray structures of CCR7, CCR2, CCR9, CXCR2, and β2AR in complex with their synthetic intracellular allosteric modulators. Cmp2105 (CCR7 — PDB: 6QZH), CCR2-RA-[*R*] (CCR2 — PDB: 5T1A), Vercirnon (CCR9 — PDB: 5LWE), 00767013 (CXCR2 — PDB: 6LFL), and Cmpd-15PA (β2AR — PDB: 5X7D) are synthetic intracellular modulators (green spheres) that bind to an allosteric binding pocket (red) on the intracellular side of the receptor that overlaps with the G-protein/Arrestin binding site (yellow). Because the binding site is occupied by a small molecule the G-protein cannot bind and therefore cannot transduce a signal towards the intracellular side. Intracellular modulators also exhibit an allosteric effect by locking the receptor in an inactive conformation that does not favor a stable interaction with endogenous chemokines.

**Figure 3.**
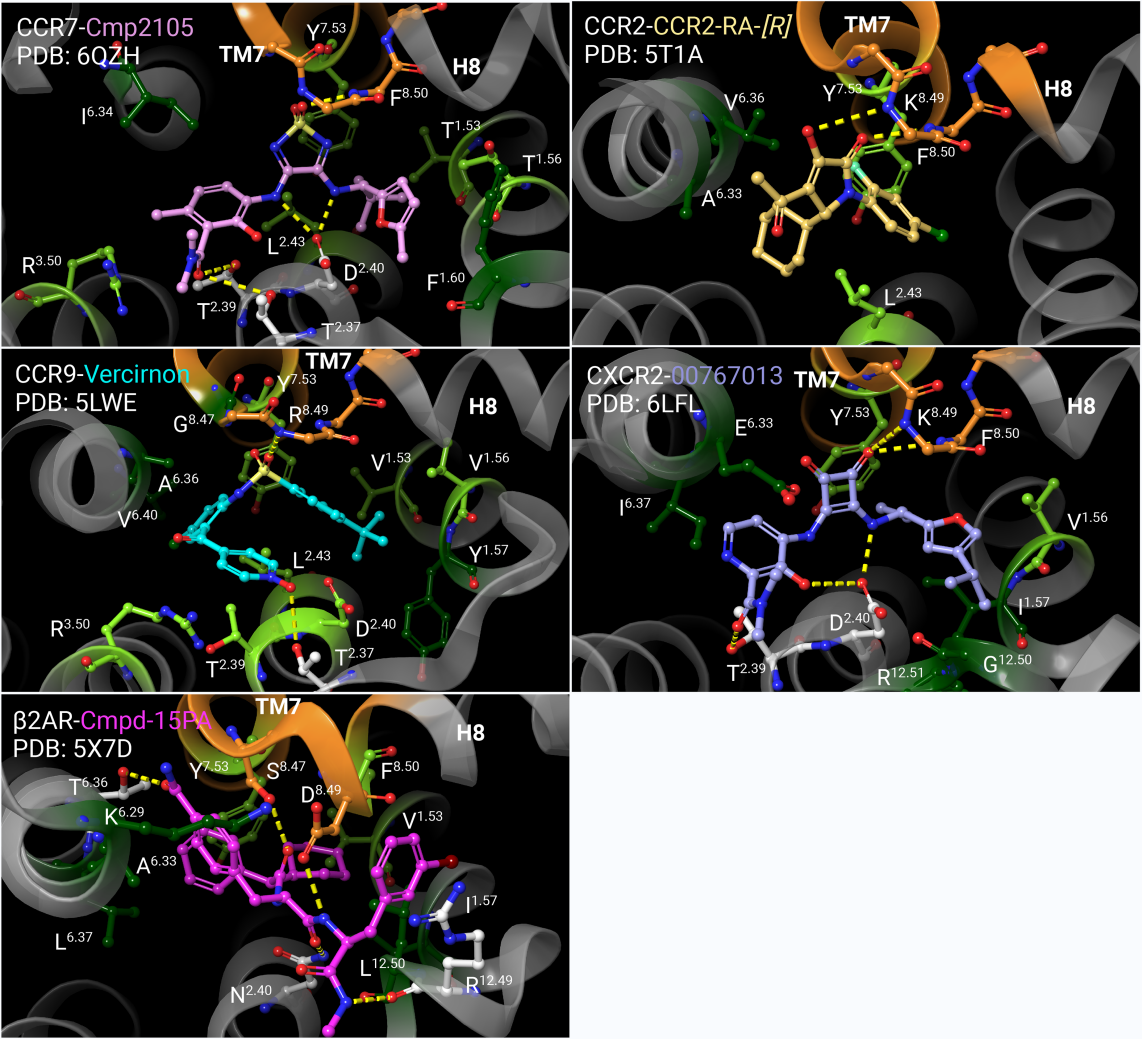
Binding mode comparison between synthetic intracellular allosteric modulators in CCR7 (upper left), CCR2 (upper right), CCR9 (middle left), CXCR2 (middle right), and β2AR (bottom left). Common for each small molecule is an interaction with a conserved motif between TM7 and Helix 8 (orange, only interacting residues are shown). Oxygen groups that are part of a thiadiazole-dioxide motif in CCR7 (PDB: 6QZH), a hydroxypyrrolinone motif in CCR2 (PDB: 5T1A), a sulfon-amide motif in CCR9 (PDB: 5LWE), a cyclobutene-dione motif in CXCR2 (PDB: 6LFL) and an amide motif in β2AR (PDB: 5X7D) facilitate hydrogen bonding (yellow lines). The interaction to the conserved motif is believed to be a key interaction to target the intracellular binding site in chemokine receptors. Hydrophobic interaction partners that are unique to the small molecule are shown in dark green. Common hydrophobic interacting residues within at least two receptors are shown in light green. Notably, Tyr^7.53^ is a common hydrophobic interacting residue in all five receptors. Residues are depicted in Ballesteros-Weinstein numbering.

**Figure 4.**
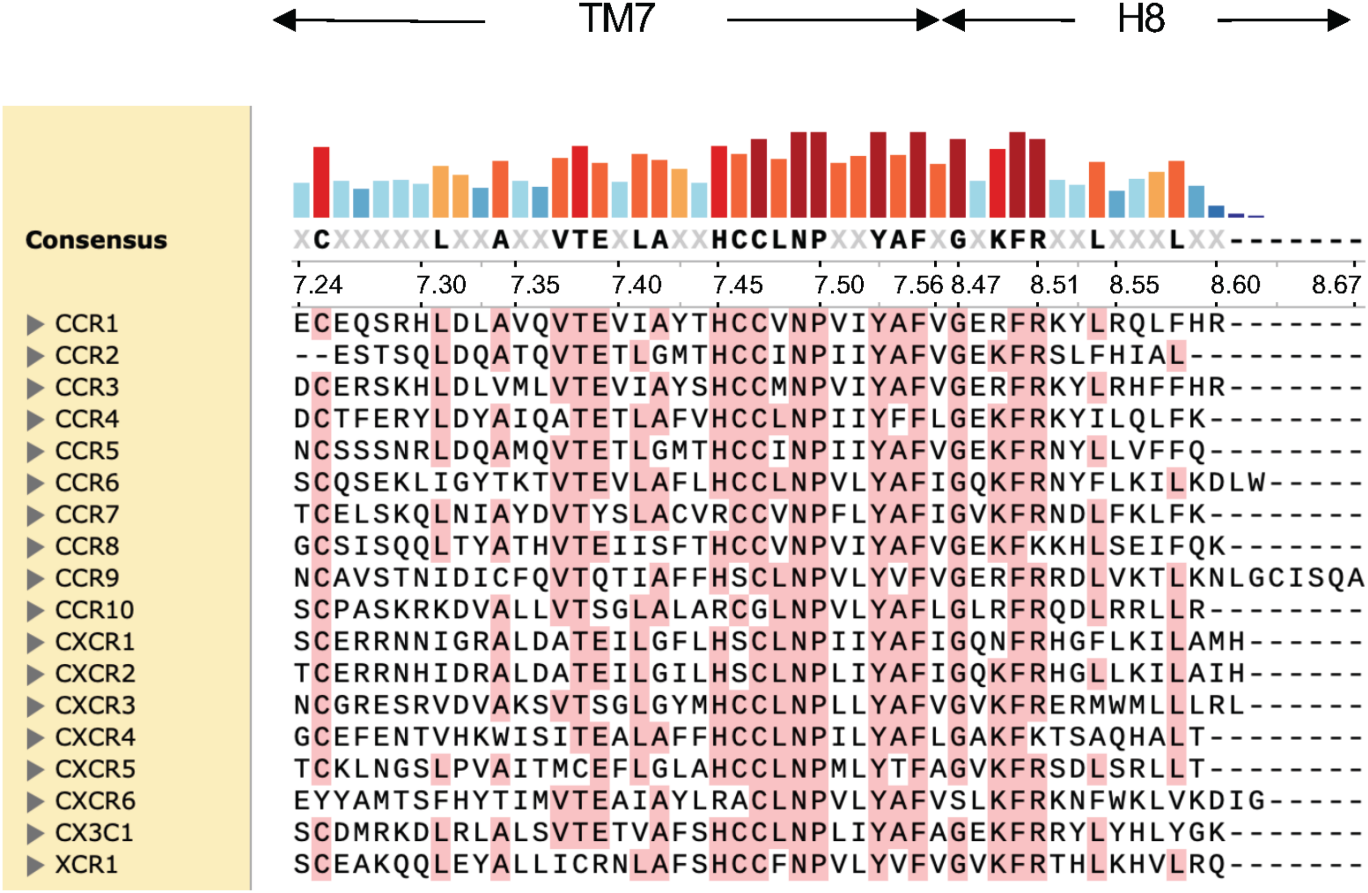
Sequence alignment of the chemokine receptor family including CCR, CXCR, and XCR receptors. The sequence alignments of TM7 and Helix 8 shows a consensus motif ranging from His/Arg^7.45^ till Arg/Lys^8.51^. Highest sequence similarity is depicted in dark red to lowest similarity in light blue. Alignment information taken from GPCRdb and prepared in SnapGene. Consensus sequences above 50% are shown.

For chemokine receptors allosteric modulators have been solely identified on the intracellular side. The concept of intracellular allosteric antagonism was initially overlooked for chemokine receptors as many small molecules were developed and described as orthosteric antagonists [16]. This perspective began to shift with the availability of crystal structures that verified the presence of small molecules on the intracellular side of chemokine receptors in the intracellular allosteric binding site. It was the structural characterization of CCR2 with the intracellular allosteric modulator CCR2-RA-[*R*] (PDB: 5T1A), CCR7 with Cmp2105 (PDB: 6QZH), CCR9 with Vercirnon (PDB: 5LWE), and more recently, CXCR2 with 00767013 (PDB: 6LFL), that gave insights into the binding mode of small molecules on the intracellular side and were pivotal in the elucidation of the dual antagonizing mechanism of intracellular modulators: Firstly, synthetic intracellular allosteric modulators induce an allosteric effect that stabilizes the receptor in an inactive conformation, thereby inhibiting the binding of orthosteric chemokines as evidenced from radioligand assays [15]. Secondly, the intracellular binding site overlaps with the G-protein/Arrestin binding site, effectively blocking the binding of transducer proteins and consequently hindering the initiation of the signaling cascade in the intracellular lumen (Figure 2). Notable between the intracellular modulators in chemokine receptors is the consistent interaction towards a conserved motif between TM7 and Helix 8 within each receptor (Figure 3): Although chemically different all small molecules interact with the backbone of Lys/Arg^8.49^ and Phe^8.50^ of the same conserved motif, which in chemokine receptors ranges from 7.45 in TM7 to 8.51 in Helix 8 (Ballesteros–Weinstein numbering [17]) [18] (Figure 4). In addition to the identical interaction towards the TM7-H8 motif, CCR7, and CXCR2 share the interactions towards Asp^2.40^ which stabilizes the core-motif of the small molecules in the binding site. Additionally, Thr^2.37^ and Thr^2.39^ play a role in facilitating hydrogen bonding in CCR7, CCR9 and CXCR2. In contrast, CCR2-RA-[*R*], apart from the interaction with the TM7-H8 motif, is not stabilized by hydrogen bonds and is mostly stabilized via hydrophobic interactions (Figure 3), which highlights the significance of the conserved TM7-H8 motif as a key interaction to target the intracellular allosteric binding site in chemokine receptors. Interestingly, Tyr^7.53^ facilitates hydrophobic interactions in all mentioned receptors. Also, residues 1.56, 2.43, and 3.50 are involved in multiple receptors to stabilize the small molecule via hydrophobic contacts. Despite the overlapping interactions, thermofluor experiments with CCR7 using the intracellular modulators CCR2-RA-[*R*] (CCR2) and Vercirnon (CCR9), as well as Reparixin and Danirixin (verified intracellular CXCR2 modulators), did not show any stabilization of CCR7, which indicates no interaction with the intracellular binding site in CCR7 [15]. Interestingly, the intracellular allosteric binding site is also verified in the beta-2-adrenergic receptor (β2AR) via the synthetic allosteric modulator Cmpd-15PA [14] (Figure 3). The interaction profile at the β2AR shows the characteristic hydrogen bond of an oxygen moiety towards the side-chain of Ser^8.47^ (PDB: 5X7D). Ser^8.47^ in β2AR is part of the same motif between TM7 and H8 as in chemokine receptors. Furthermore, Asp^8.49^ which is also part of the TM7-H8 motif hydrogen bonds an amine group and further stabilizes Cmpd-15PA. Similar to CCR7 and CXCR2 Asn^2.40^ in β2AR also interacts with the synthetic modulator through hydrogen bonding and Tyr^7.53^ is as well involved with hydrophobic interactions like in the mentioned chemokine receptors. The discovery of this binding site in a non-chemokine receptor such as β2AR indicates the potential for a universally conserved intracellular binding site across GPCRs that offers new avenues for drug discovery in class-A GPCRs.

Verified small molecule intracellular allosteric modulators center around certain key scaffolds: Urea-based (including squaramide, thiadiazole, and thiadiazole(di)oxide derivatives), sulfonamide-based, nicotinamide-based, pyrrolone-based, mercaptoimidazole-based and pyrimidine-based antagonists [19]. These scaffolds have expanded drug discovery initiatives, with numerous research teams concentrating on enhancing binding features and efficacy profiles. For example, Van Hoof et al. [20] optimized previously reported thiazolo[4,5-d]pyrimidines for CXCR2 and discovered binding to CCR7 which led then to the target optimization for CCR7 [21]. Ortiz Zacarías et al. [22,23] developed dual CCR2 and CCR5 antagonists based on triazolopyrimidinone derivatives within a multitarget approach, as well as a covalent antagonist for CCR2 based on the intracellular modulator SD-24, a sulfonamide derivative. Interestingly, the developed compounds in both studies were docked against their target receptor. The docked poses showed interactions towards the conserved TM7-H8 motif, which further suggests the pivotal role of the motif in targeting the intracellular binding site.

All of the small molecules discussed above act as antagonists through the intracellular binding site. Recently the inverse agonist Cpd 1 has been reported for the class-A receptor GPR61 within a slightly shifted binding mode within the intracellular binding site in comparison with chemokine receptors (PDB: 8TB0) [24]. Cpd 1 stabilizes as an inverse agonist the outward movement of TM6 in a wedge-like mechanism, which does not lead to GPCR activation. Astonishingly, Cpd 1 is also based on a sulfonamide scaffold and interacts with Asn345^8.47^ in GPR61 which is part of the same key TM7-H8 motif as in chemokine receptors and the β2-adrenergic receptor.

Targeting the intracellular allosteric binding site and the identification of small molecules is not trivial as shown by a comprehensive virtual screening campaign using structure- and ligand-based screenings on CCR7. Proj et al. [25] used the ZINC in-stock subset library containing 13.7 million drug-like compounds for virtual screenings. The 287 *in silico* hits were verified via a calcium signaling assay that concluded without any confirmed binders which highlights the difficulties in identifying new scaffolds and binders for this site. Nevertheless, the intracellular binding site is not only limited to small molecules. Various strategies have been proposed to exploit the unique characteristics of this site, including pepducins, antibody-fragment-based binders, intrabodies, and PROTACs [26–28]. In addition, the development of fluorescently labeled ligands for CCR2 and CCR9, based on the original allosteric modulators, further expands the toolkit to study chemokine receptors using the intracellular binding site [29,30].

In summary, the intracellular allosteric binding site provides unique opportunities for drug discovery. The binding site is not limited to chemokine receptors, thereby expanding the potential to target the site in a diverse range of receptors. Furthermore, the modulatory effects of compounds binding to this site may not be restricted to negative allosteric modulators, as observed in the case of GPR61. This broadens the scope for exploiting this binding site for the development of therapeutic interventions.

## Insights from active chemokine receptor structures

Single particle cryo-electron microscopy (cryo-EM) has significantly improved our understanding of chemokine receptor activation through the structural elucidation of 17 chemokine receptor structures (9 unique receptors) in an active state, covering all subgroups of chemokine receptors: CC, CXC, CX3C, and ACKR (Figure 1). Among these structures, twelve were solved with an agonist and G-protein, four structures depict the receptor in an apo state with a bound G-protein, and one structure showcases the receptor in an apo state with a bound fab fragment. For a comprehensive overview of active GPCR structures readers are referred to the GPCRdb (https://gpcrdb.org) [31].

In the following sections, we present an overview of the current activation mechanism model at the orthosteric site, along with structural insights into biased signaling as well as G-protein interactions.

### Orthosteric chemokine receptor activation

In this section, we will provide a summary of the latest insights into chemokine receptor activation. For an in-depth examination of activation mechanisms in chemokine receptors, we direct readers toward Gustavsson [32] and Urvas and Kellenberger [33].

**Figure 5.**
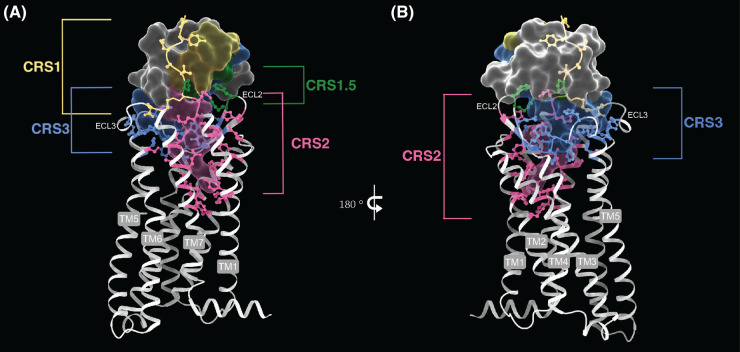
Chemokine recognition sites. Structure of CCR1-CCL15L or CCL15(26–92) (PDB: 7VL9) used to depict various chemokine recognition sites. Surface representation of bound CCL15 with all four CRSs mapped along with their respective interacting receptor residues. CRS1 and CRS2 are depicted in yellow and hot pink, respectively, and the recently defined CRS1.5 and CRS3 are depicted in forest green and cornflower blue, respectively. Chemokine binding initiates with CRS1 interacting with the N-terminus of the receptor (residues marked in yellow). CRS1.5, crucial for CCR-CCL complex stabilization, has three interactions between the ligand and receptor. CRS3 interactions extend from the N-terminus of ECL2, and β1/β2 strands of the chemokine until ECL3 of the receptor, and the 30s loop of the chemokine, covering almost a 180°. This makes CRS3 a prominent recognition site and a probable determining factor for ligand specificity. Signal propagation is induced via CRS2 with receptor residues deeper in the pocket of TM1, 2, and 7, marked in hot pink. CRS2 in chemokines is mostly composed of N-terminal residues that are proposed to interact with microswitches within the receptor, for further signal transduction towards the intracellular binding site. (B) 180° rotation shows the extent of interactions from ECL2 of the receptor with CRS3 of the chemokine.

Historically, a two-site model has been postulated to explain the multi-step activation and signaling process of chemokine receptors. The model involves two distinct and sequential interaction sites: chemokine recognition site 1 (CRS1) and chemokine recognition site 2 (CRS2) (Figure 5) [34]. CRS1 primarily involves the interaction between the N-terminus of the receptor and the N-loop/β3-strands and 40s-loop of the chemokine. These interactions are crucial for establishing binding affinity between the chemokine and its corresponding receptor. Subsequent to the initial docking at CRS1, a secondary interaction occurs at the CRS2, which involves the engagement of the chemokine's N-terminus with the receptor's deeper orthosteric binding pocket. This subsequent interaction is pivotal for receptor activation, triggering the conformational changes necessary for intracellular signaling.

More recently two additional CRS's were described which are termed CRS1.5 and CRS3 that have refined our understanding of chemokine recognition (Figure 5). CRS1.5 is situated between CRS1 and CRS2 and represents a transitional zone where elements of both binding sites converge [35]. CRS1.5 is composed of the PC-motif within the N-terminus of the receptor which is positioned opposite of the disulfide bridge between the N-loop and β3-strand of the chemokine. The interactions at CRS1.5 are thought to play a role in stabilizing the chemokine-receptor complex and assisting in the alignment of the chemokine for the subsequent engagement with CRS2. CRS3 was proposed within the structure determination of CCR1 and entails the interaction of the β1/β2 strands and the 30s loop of the chemokine with ECL2 and ECL3 of the receptor, with additional interactions with TM5 and TM6 of CCR1 [36]. CRS3 was proposed because the β1/β2 strands and the 30s loop of the endogenous chemokines for CCR1 showed a higher sequence conservation which indicates that CRS3 could be important in determining ligand specificity. Variations in CRS3 can therefore influence the binding affinity and signaling specificity of different chemokines.

Chemokines engage with their receptors through a hook-like mechanism in which the N-terminus of the chemokine interacts with the deeper part of the orthosteric binding site within CRS2. Nevertheless, through the availability of many active chemokine receptor structures differences were observed in how chemokines interact with their respective receptor. Some chemokines engage the orthosteric binding site with their N-terminus deeper, for example CCL2 in CCR2 [37] (PDB: 7XA3) and CCL3 in CCR5 [38] (PDB: 7F1Q), while others, for example CCL20 in CCR6 [39] (PDB: 6WWZ) and CXCL8 in CXCR2 [40] (PDB: 6LFM), show a very shallow binding. Within the binding site the N-termini can also adopt chemokine receptor family specific conformations as demonstrated by Ngo et al. [41]*.* Using disulfide cross-linking and molecular modeling the authors showed that the N-terminus of CXCL12 in CXCR4 adopts a unique CXC-motif-dependent conformation. The spatial arrangement of the negatively charged Asp262^6.58^ and the interaction with R8 within the ‘ELR’ motif that is unique to CXC chemokines might be the determining factor in CXCR chemokine recognition.

To further delve into the intricacies of ligand specificity we analyzed the binding interactions of the three available active CCR5 structures bound to MIP-1α (PDB: 7F1Q), RANTES (PDB: 7F1R) and 6P4[CCL5] (PDB: 7O7F). We found three common receptor interactions shared by all mentioned chemokines that are, interestingly, spatially in proximity within CRS3 which is implicated in ligand specificity: Ser^45.52^ (ECL2), Tyr^5.31^, Lys^5.35^. In addition to the shared interactions, each chemokine displayed also exclusive binding partners. Comparison with the approved anti-HIV CCR5 drug Maraviroc shows that the N-terminus of RANTES and MIP-1α share the Maraviroc interaction with Tyr^6.51^ and MIP-1α additionally the interaction with Glu^7.39^, indicating similar interaction requirements between small molecules and chemokines within the deeper end of the orthosteric binding pocket.

**Figure 6.**
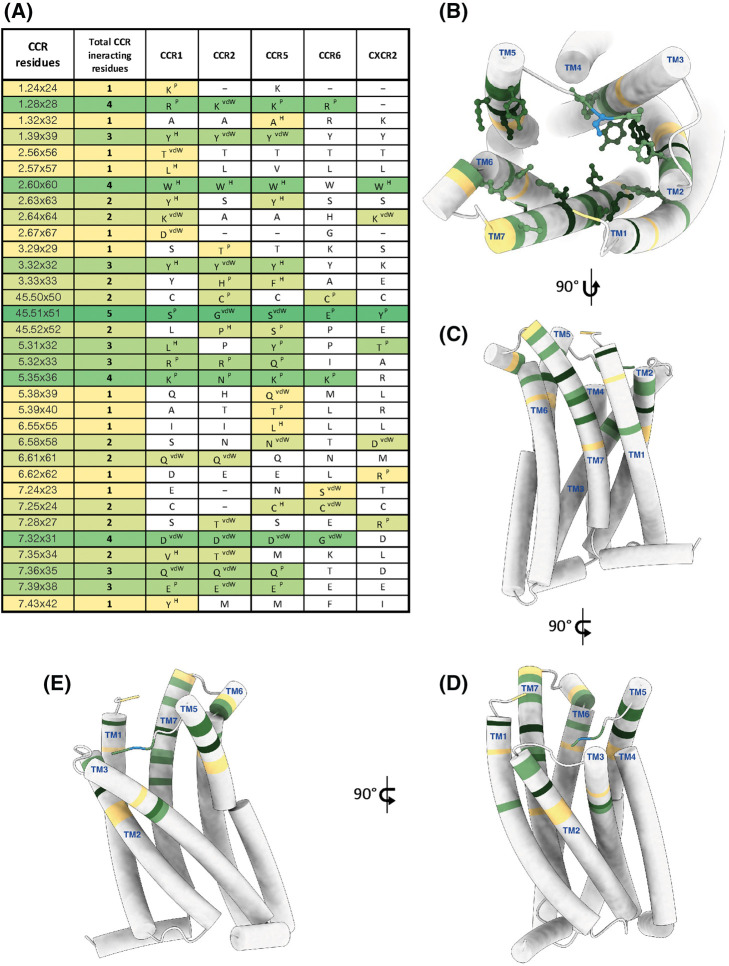
Chemokine-receptor interaction matrix. (**A**) Tabular view of receptor residues which interact with chemokines among five different CCRs. The first column (CCR residues) lists all interacting residues from TM1 till TM7. The second column (Total CCR interacting residues) summarizes the residues color-coded from highest (darkest green) to lowest interaction count (yellow) within these five CCRs. Column three to seven list the individual residues for CCR1, CCR2, CCR5, CCR6 and CXCR2. Interaction types have been shown as superscript on the interacting residues (H for Hydrogen, P for Polar, vdW for van der Waal). (**B**) Top view of the interaction cavity of all 32 residues from 5 CCRs mapped on CCR5 model (PDB: 7O7F). Only residues interacting in two or more CCRs with their respective chemokines are shown with side chains. Ser45.51 × 51 with higher consensus is shown in blue. (**C–E**) are representations of the interaction cavity from different angles showing the depth and extent of the chemokine-CCR residue interaction. Data obtained from GproteinDb.

To find common interaction patterns across various chemokine-receptor complexes we utilized the GPCRdb to characterize the interaction profile of five chemokine structures bound to their receptor (Figure 6). We analyzed the structures of CCR1 (PDB: 7VL9), CCR2 (PDB: 7XA3), CCR5 (PDB: 7O7F), CCR6 (PDB: 6WWZ) and CXCR2 (PDB: 6LFO) and identified the residue 45.51 within ECL2 to have an interaction in each receptor when considering hydrophobic, polar and van der Waals interactions. ECL2 adapts its conformation upon chemokine binding and therefore directly responds to the binding event. Mutation of residue 45.51 in CXCR4 led to a 8–14 fold decrease in the agonistic potency of CXCL12 [42]. 45.51 is part of CRS3 which, as mentioned above, is implicated to play a role in determining ligand specificity. Importantly, CRS3 surrounds the chemokine by almost 180° and includes ECL2 as well as residues of ECL3 (Figure 5). Larsen et al. [43] reported a similar important role for ECL2. The authors identified residue ‘C^45.50^ + 4’ as beeing part of a hydrophobic cluster together with residues in TM4 (4.63) and TM5 (5.34). Mutation of C^45.50^ + 4 in 12 chemokine receptors led within half of the receptors to a severely or entire impairment of the signaling, whereas the other half were largely unaffected. These findings highlight the importance of ECL2 for the chemokine activation mechanism.

Furthermore, we found that residue 1.28, the largely conserved residue 2.60, as well as 5.35 and 7.32 engage with the chemokine in four out of five receptors. While the interaction in 1.28 is mostly due to polar hydrogen bonds, 2.60 engages exclusively with hydrophobic interactions, 5.35 exclusively with polar interactions, while 7.32 is in close contact and interacts with van der waals interactions with the chemokine. Interestingly, 1.28, 2.60 and 7.32 are located in CRS2 and engage with the N-terminus of the chemokine, suggesting that these residues trigger conformational changes that lead to the active conformation of the receptor, while 5.35 is located in CRS3 and plays a role in ligand specificity.

The above-mentioned observations challenge the traditional two-site, two-step activation model. Consequently, Sanchez et al. [44] introduced a three-step model, in which the authors postulated that the sole binding interactions in CRS1 and CRS2 are insufficient for complete receptor activation. The proposed three-step model initiates with the nonspecific, low-affinity binding of the chemokine in CRS1 (step 1), followed by specific binding interaction within CRS2 with possible additional interactions (step 2), and concludes with a conformational change in the chemokine-receptor complex that leads to receptor activation and signaling (step 3). This model separates the high-affinity binding event from receptor activation.

### Biased signaling in chemokine receptors

The chemokine system displays promiscuity, as most receptors respond to multiple chemokines, and most chemokines are able to activate multiple receptors. While initially thought to be a redundant system it is now clear that chemokines exhibit biased agonism properties when interacting with their respective receptor, meaning that the triggered signaling pathway depends on the bound ligand for the same receptor [45]. Recent research shows that two factors influence the signaling outcome: (1) the interaction of the chemokine with the receptor in the orthosteric binding site triggers allosteric microswitches that either favor G-protein, Arrestin, or balanced signaling, and (2) a specific phosphorylation barcode at the C-terminus of the receptor that is unique to the bound ligand.

Shao et al. [36] demonstrated the molecular determinants of biased signaling in CCR1 by using variants of CCL15 with N-terminal truncations, termed CCL15^L^ (residues 26–92), CCL15^M^ (residues 27–92), and CCL15^S^ (residues (28–92), (31–92)). Shao and colleagues found that CCL15^L^ showed biased behavior towards Arrestin signaling, CCL15^M^ was balanced between Arrestin and G-protein signaling and the short version CCL15^S^ was strongly biased towards the G-protein pathway, suggesting that the N-terminus was a determinant of Arrestin recruitment. Structural investigation revealed that while the main interactions between CCL15^L^ and CCL15^M^ were similar, the sole determinant of signaling bias was due to conformational rearrangements of the conserved Y291^7.43^ residue. In the apo-state, Y291^7.43^ formed hydrogen bonds with T86^2.56^ and W90^2.60^ and pointed toward TM2. In contrast, the binding of CCL15^L^ led to a conformational rearrangement of Y291^7.43^ due to the breakage of the hydrogen bonding with T86^2.56^ and W90^2.60^ via the last N-terminal amino acid F26 of the chemokine. Y291^7.43^ then established interactions with Y113^3.32^ and Y255^6.51^ that favor β-arrestin recruitment in CCR1. In the case of CCL15^M^ (balanced behavior), Y291^7.43^ was found to take in two alternative conformations. One that resembles the apo state and one the CCL15^L^ conformation, hence the balanced signaling mode. Mutational experiments further proved the point. Y291A abolished the biased signaling properties of the receptor when activated with CCL15^M^ and CCL15^S^, highlighting its importance for biased behavior in CCR1.

Eiger et al. [46] showed that the CXCR3 chemokines CXCL9, CXCL10, and CXCL11 direct distinct phosphorylation patterns at the C-terminus of the receptor that lead to discrete biological functions. For example, CXCL9 binding significantly increased the abundance of the phosphopeptide DSSWSETSEASYpSGL (S366), while CXCL10 and CXCL11 did not. Additionally, the different phosphorylation ensembles influenced G-protein activation, β-arrestin recruitment, as well as receptor internalization rates and therefore directly influenced the signaling outcome directed by the chemokine.

These results show that the chemokine system is far from being a redundant system in a classical sense. Each chemokine not only induces specific conformational arrangements within the receptor but also stimulates distinct phosphorylation patterns that further contribute to an immensely fine-tuned response. The elucidation of these nuanced modifications paves the way for the development of biased therapeutics that offer the promise of enhanced safety and minimized side effects. The case of CCR1 exemplifies how basic research can significantly influence drug discovery endeavors. Although CCR1 has been identified as a potential target for the treatment of autoimmune and allergic disorders, drug development efforts have met with limited success. However, by elucidating the role of Y291^7.43^ within biased signaling in CCR1, it is now feasible to leverage this knowledge for drug discovery initiatives. For instance, disrupting the interaction between Y291^7.43^, T86^2.56^, and W90^2.60^ using small molecules or peptides, while simultaneously stabilizing the interaction between Y113^3.32^ and Y255^6.51^, could serve as a promising approach to selectively activate β-arrestin signaling pathways. Moreover, the discovery of biased agonism in CCR1 raises the possibility that similar mechanisms may be present in other chemokine receptors, thereby unlocking new opportunities for drug discovery across the entire chemokine system.

### Intracellular G-protein binding in chemokine receptors

**Figure 7.**
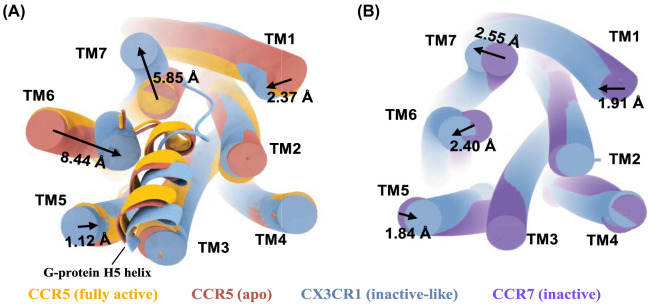
Comparison of G-protein binding in CCR5 (full active), CCR5 (apo) and CX3CR1. (**A**) Comparison of the G-protein bound structures reveals that the G-protein in the CCR5 apo structure (red, PDB: 7F1S) is almost identical positioned compared with the fully active CCR5 structure (yellow, PDB: 7F1Q). CX3CR1 shows significant TM6 inward (8.44 Å) and TM7 outward (5.85 Å) movement, typical for inactive-state structures. The C-terminus of the G-protein in the fully active CCR5 (yellow), as well as in the CCR5 apo structure (red) and CX3CR1 structure (blue) are shown in helix form. CCR1 apo and CCR3 apo structures are not shown as they are in an identical position as the CCR5 apo structure. The apo state CCR5 and inactive-like CX3CR1 (blue) are superposed on the fully activated CCR5 (yellow). (**B**) Comparison between CX3CR1 and CCR7 (inactive). CX3CR1 (blue) is in a similar position as the inactive state CCR7 (purple) with a slight further outward position of TM6 and TM7 (2.40 Å and 2.55 Å, respectively). Intracellular loops and helix 8 are removed for a clearer view.

The activation of chemokine receptors involves the binding of a chemokine from the extracellular side and the heterotrimeric G-protein from the intracellular side, leading to a fully active conformation of the receptor. This active conformation is typically characterized by an outward movement of TM6 and an inward movement of TM7, which accommodates the C-terminal helix (H5) of the G-protein on the intracellular binding site. Chemokine receptors have been solved as fully active complexes consisting of the receptor, the chemokine, and a G-protein, as well as in apo states without a chemokine in the orthosteric binding site. Interestingly, the apo structures of CCR5 (PDB: 7F1S), CCR1 (PDB: 7VL8), and CCR3 (PDB: 7X9Y) resemble a canonical active conformation on the intracellular side, with the G-protein taking in an almost identical position (Figure 7A). However, on the extracellular side the apo structures differ from each other due to a non-stabilized binding site, as indicated by generally higher b-factors.

In contrast, the recently solved CX3CR1 structures, as a GPCR complex (PDB: 7XBX) and in apo form (PDB: 7XBW), show a very different arrangement of the helices on the intracellular side [47]. Both CX3CR1 structures appear in an inactive-like conformation despite the presence of a bound G-protein. Compared with canonical active structures, TM6 of CX3CR1 shows an inward movement of 8.44 Å and TM7 shows an outward movement of 5.85 Å (Figure 7A). Lu et al. attribute this unusual conformation to the presence of three cholesterol molecules that may stabilize the inward conformation of TM6. Furthermore, key residues such as R^3.50^ of the DRY motif, the highly conserved W^6.48^ within the CWxP motif, and the PIF/Y motif resemble inactive-like conformations. The CX3CR1 structures more closely align with the inactive CCR7 structure (PDB: 6QZH) in which TM6 and TM7 only change the position by 1.50 Å and 1.10 Å respectively (Figure 7B). Additionally, due to the inward shift of TM6 in CX3CR1, the G-protein H5 C-terminus is oriented towards TM7 and Helix 8 and therefore does not resemble the canonical G-protein interaction pattern (Figure 7A).

In summary, while most chemokine receptor structures reveal a consistent pattern of conformational changes associated with activation, the CX3CR1 structures challenge this paradigm by exhibiting an inactive-like conformation despite the presence of a bound G-protein. This unique structural arrangement raises questions about the complex activation mechanism of chemokine receptors and highlights the need for further investigation to fully understand the implications of these findings. On the other hand, the apo-state structures show canonical G-protein binding which may indicate the intrinsic tendency of these receptors to adopt an active-like conformation and potentially facilitate rapid activation upon chemokine binding or having basal activity.

## Conclusions

The elucidation of chemokine receptor structures has significantly advanced our understanding of their signaling mechanisms and opened novel therapeutic pathways. Figure 1 illustrates this progress and shows the initial period from 2010 to 2019 that focused on inactive structures through X-ray crystallography and the subsequent shift to single-particle cryo-EM that enabled the exploration of active chemokine receptor structures.

Initially, the chemokine system was perceived as a redundant system in which for example the binding of two chemokines for the same receptor would result in the same signaling outcome. Recent experimental and structural insights reveal a very fine-tuned system in which ligand-, receptor- and tissue bias leads to a nuanced orchestration of cellular responses that is crucial for maintaining biological functions. The expansion of the chemokine recognition two-site model to include the additional chemokine recognition sites 1.5 and 3 aims to reconcile these recent insights to provide a more comprehensive explanation for ligand specificity and biased signaling events in chemokine receptors. These new findings may be accommodated within the term ‘integrated multisite activation model’ that emphasizes that receptor activation is not a result of isolated interactions at individual sites within defined steps. Instead, it is the culmination of a network of interactions across multiple sites and involves a series of interconnected steps: CRS1 facilitates the initial contact with the chemokine, establishes binding affinity, and subsequently guides the chemokine towards the orthosteric binding pocket. The subsequent interaction with CRS1.5 stabilizes the chemokine-receptor complex and assists in the alignment of the chemokine towards CRS2 in which the main interactions occur that trigger the conformational changes that are necessary for G-protein binding. Ligand specificity is thought to be influenced by CRS3 which includes ECL2 that emerges as an important player in chemokine recognition. We identified the ECL2 residue 45.51 as the only common interacting residue in all analyzed structures. Mutational studies of 45.51 diminish chemokine signaling which signifies alongside the previously reported ‘C^45.50^ + 4’ residue in ECL2 the importance of the extracellular loop in the chemokine activation mechanism.

Significant progress has been made in understanding biased signaling in chemokines receptors. Y291^7.43^ in CCR1 is shown to be a pivotal residue in determining the signaling pathway. Distinct phosphorylation patterns at the C-terminus for each interacting chemokine highlight a very fine-tuned system.

Allosteric modulation, on the other hand, at intracellular sites is equally interesting as it offers novel mechanisms of receptor inhibition which might not be limited to chemokine receptors. Allosteric modulators that interact with the intracellular binding site can selectively modulate receptor activity and therefore potentially allow for more precise control compared with traditional orthosteric drugs. Allosteric modulators do not compete with natural ligands for the same binding site and therefore offer several advantages compared with traditional drugs.

Taken together the recent advancements in elucidating the structural details of chemokine receptor signaling and modulation represent a significant leap in our fundamental understanding of these receptors. These advancements open up new avenues for the development of more targeted and effective therapeutics for a wide range of immune and inflammatory-related disorders.

## Perspectives

- A deep comprehension of signaling mechanisms are vital for elucidating how cellular communication networks are regulated, especially for chemokine receptors that facilitate the body's immune response and guide cellular movements. Understanding these complex interactions paves the way for innovative therapeutic strategies. Precise manipulation of these signaling pathways hold an immense potential for clinical application to treat a wide range of diseases.
- The complementary application of X-ray crystallography and single-particle cryo-EM has yielded profound structural insights into inactive and active chemokine receptor states. The discovery and verification of the intracellular binding site in chemokine receptors has unveiled numerous opportunities for leveraging this site for therapeutic purposes. Moreover, investigating active receptor structures has significantly enhanced our view of chemokine binding and receptor activation mechanisms, as well as unveiled the nuances of signaling bias in chemokine receptors.
- Investigating the intracellular binding sites across class-A receptor families and targeting GPCRs from the intracellular site offer a promising avenue for drug discovery and a novel strategy to modulate GPCR activity. Additionally, further research is essential to understand the function of residue 7.43 in various receptors and its contribution to biased signaling. Although G-protein structures associated with class-A and chemokine receptors are widely studied, Arrestin structures remain relatively underexplored. Moreover, current structural studies primarily focus on isolated proteins; hence, adopting an *in situ* approach to examine chemokine receptors within a cellular context might be crucial for advancing our understanding of their physiological roles. Time-resolved studies can complement this approach on a protein level to further pinpoint chemokine recognition and activation mechanisms.

## Abbreviations

ACKR: atypical chemokine receptors
CRS1: chemokine recognition site 1
CRS2: chemokine recognition site 2
cryo-EM: cryo-electron microscopy
GPCR: G-protein coupled receptor
